# How to Prepare Spectral Flow Cytometry Datasets for High Dimensional Data Analysis: A Practical Workflow

**DOI:** 10.3389/fimmu.2021.768113

**Published:** 2021-11-19

**Authors:** Hannah den Braanker, Margot Bongenaar, Erik Lubberts

**Affiliations:** ^1^ Department of Rheumatology, Erasmus University Medical Center, Rotterdam, Netherlands; ^2^ Department of Immunology, Erasmus University Medical Center, Rotterdam, Netherlands; ^3^ Department of Clinical Immunology and Rheumatology, Maasstad Hospital, Rotterdam, Netherlands

**Keywords:** spectral flow cytometry, machine learning, workflow, data analysis - methods, R

## Abstract

Spectral flow cytometry is an upcoming technique that allows for extensive multicolor panels, enabling simultaneous investigation of a large number of cellular parameters in a single experiment. To fully explore the resulting high-dimensional single cell datasets, high-dimensional analysis is needed, as opposed to the common practice of manual gating in conventional flow cytometry. However, preparing spectral flow cytometry data for high-dimensional analysis can be challenging, because of several technical aspects. In this article, we will give insight into the pitfalls of handling spectral flow cytometry datasets. Moreover, we will describe a workflow to properly prepare spectral flow cytometry data for high dimensional analysis and tools for integrating new data at later time points. Using healthy control data as example, we will go through the concepts of quality control, data cleaning, transformation, correcting for batch effects, subsampling, clustering and data integration. This methods article provides an R-based pipeline based on previously published packages, that are readily available to use. Application of our workflow will aid spectral flow cytometry users to obtain valid and reproducible results.

## Introduction

Over the years, the number of variables measured in flow cytometry experiments has increased, especially with the recent development of spectral flow cytometry. Not being limited to the number of channels of the instrument, spectral flow cytometry enables multicolor panels with many more parameters than ever deemed possible in conventional flow cytometry. Up to date, panels of over 40 colors have been developed (1, 2), with larger panels expected to emerge in the near future. Even in conventional flow cytometry, technologies are used to increase the number of markers included in one panel (3). With the current complexity of flow cytometry assays, reproducibility is a major concern. Adherence to established general guidelines for key practical aspects and data analysis will help to increase reproducibility (4). However, easy-to-use data analysis workflows for spectral flow cytometry, for the starting researcher in this field, are currently limited.

With the increase in number of markers, especially in spectral flow cytometry datasets, flow cytometry data have become high-dimensional. Therewith, traditional 2D manual gating strategies fail to comprehend and depict the entirety of the data, since the number of 2D plots increases exponentially with the number of parameters measured. Moreover, manual gating strategies limit exploration of the data. Therefore, unbiased exploration of data using automated analyses can help find unknown cell populations and can help compare cell populations between groups in a more reproducible way.

In recent years, automated analysis methods have been proven useful in multicolor flow cytometry as well as mass cytometry single cell data (5, 6). Examples of such analyses are t-distributed stochastic neighbor embedding (t-SNE) (7), hierarchical stochastic neighbor embedding (h-SNE) (8), and uniform manifold approximation and projection (UMAP) (9). These algorithms reduce dimensionality of the data and enable easy visualization of high-dimensional data. Clustering algorithms, such as spanning-tree progression analysis of density-normalized events (SPADE) (10) and flow cytometry self-organizing map (FlowSOM) (11), can visualize the data, but also cluster data. This is useful for statistical testing comparing median expression of number of cells per cluster and/or group, which is not possible in standard t-SNE or UMAP. Some of these tools were also integrated in current flow cytometry software, such as FlowJo™ software and Cytobank, or new visualization software, such as Cytosplore (12, 13).

Despite the need for automated analyses, laboratories specialized in flow cytometry often lack bioinformatics expertise and tend to stick to traditional manual gating. Not knowing where to start, new analysis strategies can be intimidating to work with and mistakes are easily made. Furthermore, specific characteristics of spectral flow cytometry, such as removal of auto-fluorescence per cell, higher maximum fluorescence intensities and minimal requirement for spectral compensation, also hinder easy use of already published workflows for data analysis in mass cytometry or conventional flow cytometry (14, 15).A few groups published advanced workflows also suited for spectral flow cytometry (16–18). Commercial flow cytometry software, such as Cytobank, also provide automated pipelines, such as CITRUS (19). However, easy step-by-step data analysis plans for researchers starting with spectral flow cytometry are lacking.

This article provides points of attention when handling spectral flow cytometry datasets and describes a workflow on how to properly prepare them for automated analysis. If needed, the workflow can also be adapted to work with conventional or mass cytometry data. The R based pipeline is based on previously published R packages, that are publicly available to use, and additional code is provided in this article. Using multiple R packages increases functionality and flexibility in the data analysis. New data from 31-color spectral flow cytometry on peripheral blood mononuclear cells from healthy controls (publically available FR-FCM-Z4KT) and a publicly available spectral flow cytometry dataset (FR-FCM-Z3WR) are used to demonstrate the workflow step by step, providing any researcher the tools to apply automated analysis on their own spectral flow cytometry data. R code and other data used is available at https://github.com/HdBraanker/Spectral_Flow_Workflow.

## Materials and Methods

### Subjects

To obtain healthy peripheral blood mononuclear cells (PBMCs), blood was drawn from healthy volunteers. Subjects had no history of auto-immune disease, were above 18 years of age and gave a written informed consent. This study was approved by the medical ethics committee of the Erasmus MC Rotterdam.

### Isolation of Peripheral Blood Mononuclear Cells

Peripheral blood mononuclear cells (PBMCs) were extracted from heparinized blood using density gradient medium Ficoll-Paque PLUS (GE Healthcare), frozen in Roswell Park Memorial Institute (RPMI) 1640 medium supplemented with 10% dimethyl sulfoxide (DMSO; Sigma-Aldrich) and 20% heat inactivated fetal calf serum (HI-FCS; Gibco, Thermofisher Scientific) and stored in liquid nitrogen until use.

### Flow Cytometry Staining Procedure

PBMCs were thawed using RPMI supplemented with 20% FCS per standard protocol. Cells were resuspended 2 million cells per sample in 150µl FACS buffer (PBS supplemented with 0.5% bovine serum albumin and 0.05% sodium-azide). To block Fc receptor binding, cells were incubated with Human TruStain FcX™ (BioLegend, San Diego, California, USA) for 5 min at room temperature, following manufacturer’s instruction. The first monoclonal antibody (mAb) cocktail for surface staining with a total volume of 25µl, prepared with brilliant ultraviolet, brilliant violet and brilliant blue antibodies in Brilliant Stain Buffer Plus (BD Biosciences, San Jose, California, USA) was added to the cells and incubated for 15 min at room temperature. See **Table 1** for a list of the mAbs used in this study. Cells were washed once with FACS buffer before the second mAb cocktail was added, containing all other antibodies for surface staining in FACS buffer in a total volume of 25µl, and incubated for 15 min at room temperature. Cells were washed with FACS buffer and with PBS before incubation with viability staining for 15 min at room temperature. After viability staining, cells were washed with FACS buffer and prepared for intracellular staining. Cells were fixed and permeabilized using the FoxP3 Staining Buffer Set (eBioscience, San Diego, California) according to manufacturer’s protocol. Cells were then stained for intracellular targets with the third mAb cocktail in the kit’s permeabilization buffer. After incubation, cells were washed with permeabilization buffer and FACS buffer and measured on the 5-laser Aurora spectral flow cytometer (Cytek Biosciences, Fremont, California, USA). Before the use within this panel, all antibodies were titrated individually according to standard practice (4, 14).

**Table 1:** List of antibodies and viability dye used in 31-color spectral flow cytometry panel.

No.	Excitation laser	Fluorochrome	Marker	Clone	Manufacturer	Catalog no.	mAb mix	Reference controls
1	355nm/UV	BUV395	CD8	RPA-T8	BD	563795	Surface 1	Cells
2		Zombie UV	Viability		BioLegend	423108	Viability	Cells
3		BUV496	CD4	RPA-T4	BD	741134	Surface 1	Cells
4		BUV563	CD161	HP-3G10	BD	749223	Surface 1	Beads
5		BUV615	CD14	M5E2	BD	751150	Surface 1	Cells
6		BUV661	CD69	FN50	BD	750213	Surface 1	Beads
7		BUV737	TCRgd	11F2	BD	748533	Surface 1	Cells
8		BUV805	CD56	NCAM162	BD	749086	Surface 1	Cells
9	405nm/V	BV421	CCR6	G034E3	BioLegend	353408	Surface 1	Cells
10		BV480	CCR4	1G1	BD	746361	Surface 1	Beads
11		BV510	CD45RA	HI100	BioLegend	304142	Surface 1	Cells
12		BV570	HLA-Dr	L243	BioLegend	307638	Surface 1	Beads
13		BV605	Ki-67	Ki-67	BioLegend	350522	Intracellular	Beads
14		BV650	CXCR3	G025H7	BioLegend	353730	Surface 1	Beads
15		BV711	CD45RO	UCHL1	BioLegend	304236	Surface 1	Cells
16		BV750	CXCR5	J252D4	BioLegend	356942	Surface 1	Beads
17		BV785	CCR7	G043H7	BioLegend	353230	Surface 1	Cells
18	488nm/B	BB515	CD25	2A3	BD	564467	Surface 1	Cells
19		Spark Blue	CD3	SK7	BioLegend	344852	Surface 2	Cells
20		PerCP	CD19	HIB19	BioLegend	302228	Surface 2	Cells
21		BB700	CD49b	AK-7	BD	746009	Surface 1	Beads
22		PerCP-eF710	CD127	EBioRDR5	eBioscience	46-1278-42	Surface 2	Cells
23	561nm/YG	PE	FOXP3	PCH101	eBioscience	12-4776.42	Intracellular	Beads
24		PE-Dazzle594	TIGIT	A15153G	BioLegend	372715	Surface 2	Beads
25		PE-Cy5	GITR	621	BioLegend	311608	Intracellular	Beads
26		PE-Cy7	PD-1	EH12.2H7	BioLegend	329918	Surface 2	Beads
27	640nm/R	APC	CCR10	314305	R&D	FAB3478A	Surface 2	Cells
28		eFluor660	CTLA4	14D3	eBioscience	50-1529-49	Intracellular	Beads
29		APC-R700	LAG-3	T47-530	BD	565774	Surface 2	Beads
30		APC-Fire750	ICOS	C398.4A	BioLegend	313536	Surface 2	Beads
31		APC-Fire810	CD27	QA17A18	BioLegend	393214	Surface 2	Cells

### Preparation of Reference Controls

As reference controls, an unstained sample and, for every color, a single-stain reference control were acquired. Single-stain reference controls were either single-stain PBMCs or single-stain beads (UltraComp eBeads™, Invitrogen, ThermoFisher Scientific). For reference controls on PBMCs, 2 million cells were stained in 25µl of FACS buffer, Brilliant Stain Buffer Plus or permeabilization buffer, depending on which mAb cocktail the antibody belongs to, simultaneously with sample staining. 50% of cells used as reference control for viability staining were heat-killed before starting the staining procedure, to ensure sufficient signal. For controls on beads, one drop of beads (ca. 50µl) was diluted with 50µl of buffer and 1 µl of antibody was added. All reference controls underwent the exact same protocol as fully stained samples, including washes, buffers used and fixation and permeabilization steps. Reference controls were acquired once and used for unmixing of the multiple batches, since runs took place within three months and used reagents carried the same lot numbers as reference controls. For an overview of which controls were measured on beads and which controls were measured on cells, see **Table 1**.

### Data Acquisition And Manual Analysis of Spectral Flow Cytometry Data

Data were acquired and unmixed using SpectroFlo^®^ v2.2.0.3 software (Cytek Biosciences, Fremont, California, USA), using the same instrument settings every run. Every run, two of the same quality control samples were acquired to correct for batch effects later on (see section 3.4). Resulting unmixed fcs files were analyzed using manual gating in FlowJo v10.7 software (BD Biosciences, San Jose, California, USA) according to the gating strategy depicted in **Supplemental Figure 1** for demonstrative purposes.

### Data and Code Availability

R code is available at https://github.com/HdBraanker/Spectral_Flow_Workflow. FCS files are available from Flow Repository, FR-FCM-Z4KT. The second data set used was downloaded from Flow Repository, FR-FCM-Z3WR. This study was performed in the context of Covid-19 research and included a 36-color spectral flow cytometry panel (20). Only the healthy controls of this study were included for the pipeline demonstration.

## Results

**Figure 1:**
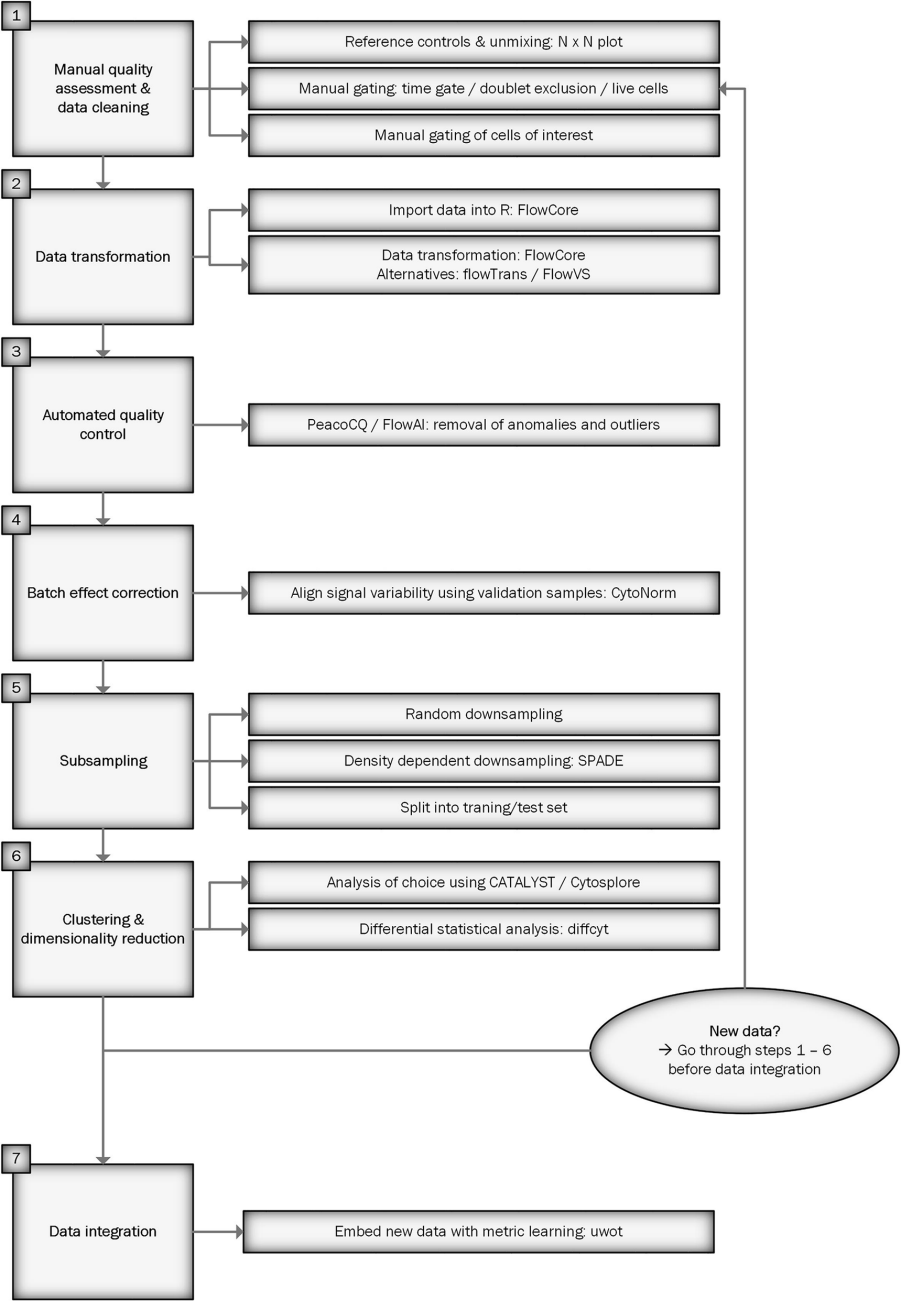
Flowchart of spectral flow cytometry workflow.

**Figure 1** shows a flowchart of the workflow for preprocessing spectral flow cytometry data for downstream analysis. We will describe each step in detail below. An R script of the workflow can be found at https://github.com/HdBraanker/Spectral_Flow_Workflow.

### Manual Quality Control and Pregating of Spectral Flow Cytometry Data

The first requirement for high-quality spectral flow cytometry data is a well-designed and titrated staining panel. This element is not covered in this article, since clear panel design guidelines already exist for spectral flow cytometry, as described in several flow cytometry guidelines (4, 14). The principles behind panel design and antibody titration in spectral flow cytometry are similar to conventional flow cytometry.

Before starting any computational method, a manual data check is required to clean up the files and to ensure exclusion of technical artifacts and bad quality samples, such as clogs, doublets and samples with low viability. This check follows the principle of “garbage in = garbage out”: analysis of low-quality data will lead to unreliable results. Underneath, manual quality control and pregating steps are described.

**Figure 2:**
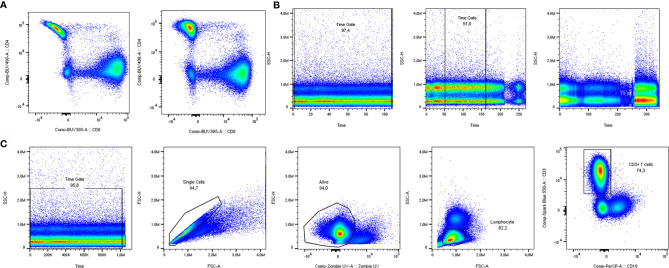
Manual quality control and data cleaning. **(A)** Unmixing results using single stain reference controls for CD4 on beads (left) vs. cells (right). **(B)** Examples of time gates where all cells could be included (left), the best part was included (middle) and a sample was excluded from analysis due to inconsistent flow rate (right). **(C)** Pregating strategy to include solely clean T cell data in downstream analysis.

A crucial step in spectral flow cytometry is unmixing of the raw data, using single-stain controls as references. Cytek’s SpectroFlo^®^ software provides a good and user-friendly unmixing function, which we used for this article. As a rule of thumb, single-stain controls on your cell type of interest give the best unmixing results. The exception is when a parameter is very lowly expressed, since the cells will lack sufficient positive signal to be a reference, making a single-stain control on beads the better option. In both cases, the same staining procedure including the same buffers should be used as when staining the full panel. To get an overview of which markers need optimization, building an N x N plot is recommended. In an N x N plot, all markers are plotted against all other markers, to check if any unmixing issues occur. As shown in **Figure 2A**, it pays off to invest time in optimization of single-stain controls. In this example, you can see a banana-shaped population when unmixing with reference controls on beads, that is corrected when cells are used as single stained reference controls. Minimal adjustments (<3%) can be made to correct for artifacts with compensation, but manual compensation should be avoided.

Having the properly unmixed and (if necessary, compensated) files, the data are prepared for manual gating. Before proceeding to automated analyses, several manual gating steps are required for quality control and cleaning of the data (17). The first of these steps is the time gate, to exclude noise due to inconsistencies in the flow rate (**Figure 2B**). Next, doublets and dead cells should be excluded. In case of low viability or lack of a consistent flow rate window, samples should be excluded from analysis to prevent skewed results. In our case, analyzing T cells from cryopreserved PBMCs, a viability of at least 80% of acquired cells was chosen, but cut-offs can depend on cell types and markers used. Lastly, there is the possibility to gate your main population of interest to include in your automated analyses. The panel used in our own dataset was focused on T cells. Therefore, manually pregated CD3+ T cells (time gate/single cells/living cells/lymphocytes/CD3+) were saved as new FCS 3.1 files (**Figure 2C**).

### Importing and Transforming Spectral Flow Cytometry Data

**Figure 3:**
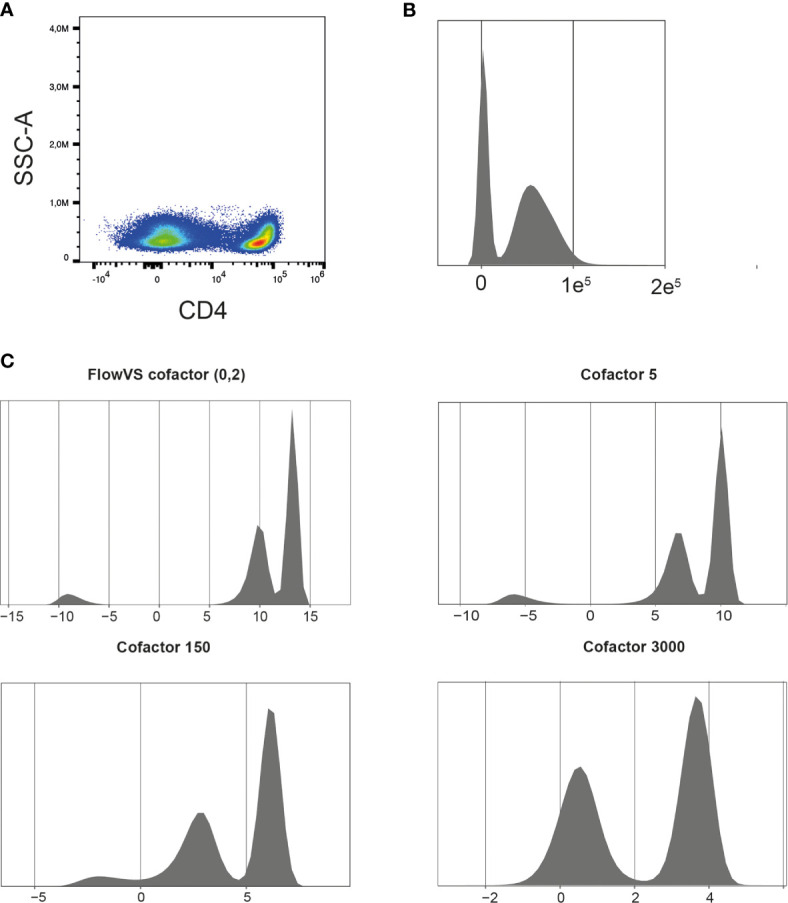
Finding the right cofactor for arcsinh transformation. **(A)** Representative CD4 pseudocolor plot. **(B)** Representative density plot of untransformed CD4 expression. **(C)** Density plots of CD4 expression after arcsinh transformation with different cofactors. Cells were pre-gated on living CD3+ T cells.

The exported FCS 3.1 files can be imported into the R environment with the FlowCore package (21). FCS3.1 files include the cell measurements and metadata. There are a few points to consider when importing spectral flow cytometer FCS files with the FlowCore package. Firstly, avoid truncation of the data when importing the files. The FlowCore package was mainly built for conventional flow cytometry and truncates data with extreme positive fluorescence intensity values. However, some spectral flow cytometers, such as Cytek Aurora, can have maximum fluorescence intensities of 4.10^6^ (14). The FlowCore package structures data and extracts expression values for all markers and cells from an FCS file. The package uses standard cut-offs for the maximum range of an expression value per marker. For spectral flow cytometry these maximum expression values can exceed those cut-offs and FlowCore truncates these values. The *Read.FCS* and *Read.FlowSet* functions provide options to prevent data truncation, also shown in our available R script. Secondly, similar to other fluorescence measurements, such as conventional flow cytometry and microarrays, spectral flow cytometry is subjected to inhomogeneity of the signal variance. The physical process of exciting, emitting and detecting fluorescence signals on a flow cytometer results in increased variance of the fluorescence signal when the mean fluorescence intensity increases (15). Data transformation will help stabilize this variance between cell populations, which helps to discriminate between cell populations and improves clustering results. Moreover, “classical” flow cytometry software, such as FlowJo and Cytobank, always show logical/bi-exponential transformed data in their 2D plots. These transformations have helped us for decades to visualize and interpret flow cytometry data. However, for computational analysis also other methods, such as generalized Box-Cox or arcsinh transformation, can be used (22). The FlowCore package provides functions to apply the different transformations, but it is recommended to also try other packages, such as flowTrans or FlowVS package (22, 23), to find the best transformation for your data. Knowledge of the biology can guide the choice for transformation and the fitness for your data. For example, in our T cell data we would expect a clear CD4 positive and CD4 negative population as is visualized by FlowJo software with a biexponential transformation (**Figure 3A**). **Figure 3B** shows a density plot made with the FlowVIZ package of the untransformed data (21). We used the arcsinh transformation, as it is widely used for computational analysis of flow cytometry data (22). The FlowVS package can calculate individual cofactors per fluorochrome for an arcsinh transformation of the data. The cofactor is the parameter that will stabilize within-population variances (23). In mass cytometry a fixed cofactor for all channels between 5 and 15 is often appropriate. In conventional and spectral flow cytometry, an individual cofactor per channel/fluorochrome is recommend, usually between 10 and 10.000 (18). However, calculating individual cofactors can be time-consuming and if fast exploration of data is needed, a fixed cofactor can temporarily be used. A good cofactor should produce two clear peaks and preferably a peak for the negative value around zero. **Figure 3C** shows the different cofactors and their effect for the CD4 marker. Cofactor 3000 shows no gaps around zero and produces two clear peaks as opposed to the other options.

### Automatic Quality Control of Flow Cytometry Data

Another preprocessing step is to remove anomalies in flow rate, signal acquisition and outlier events. Flow rate can already be checked in the manual checks and manually gated, using the time gate as described before. Different packages for cleaning flow cytometry data are available in Bioconductor, such as FlowAI, FlowClean and PeacoQC. The FlowAI package can remove anomalies for flow rate, signal acquisition and outlier events (24). FlowAI uses algorithms based on generalized extreme studentized deviate tests and binary segmentation, which make it quite strict. A large advantage of FlowAI is that it also provides an interactive way to remove anomalies. Alternatively, the PeacoQC package can be used. The PeacoQC package uses algorithms to remove anomalies based on position of cells in an isolation tree (IT) and mean absolute deviation distance (MAD). Parameters can be fine-tuned on a few samples making it easy-to-use in large datasets. The strength of PeacoQC package is mainly in samples with for example two clogs that disturbed flow rate and signal acquisition as it can select multiple disjointed regions for removal (25).

### Batch Effects

A large advantage of spectral flow cytometry machines, such as the Cytek Aurora, is that reference controls can be reused saving valuable time. However, differences between sample batches is unavoidable and will occur due to different technical causes (26). Sample handling, staining procedure and instrument performance can all cause batch-to-batch technical variability. Multiple approaches can be used to reduce batch effects. Taking technical replicates, meaning that the same sample is measured in every batch, gives great insight in technical variability between batches. However, for large (clinical) studies this method might not be feasible. Randomly distributed samples of different groups over different batches will help to reduce the influence of batch effects on the data analysis. When using fresh patient samples, that also might not be feasible. If you label your samples per batch, it is easy to check if different batches affect for example clustering, and will help interpret the results.

**Figure 4:**
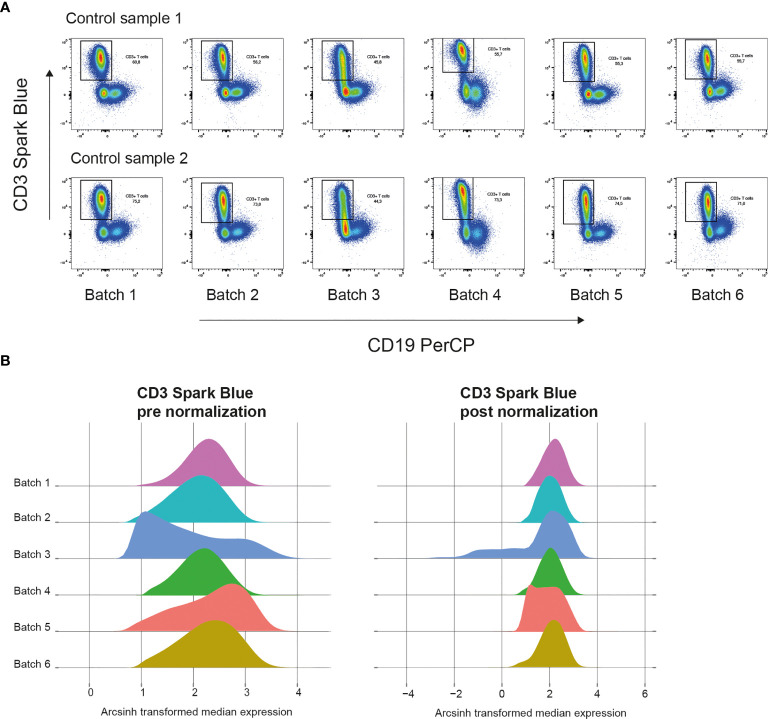
Correcting batch effects in spectral flow cytometry. **(A)** Pseudocolor plots of CD3 Spark Blue vs CD19 PerCP expression of two control samples in 6 batches measured at different dates. **(B)** Representative density plots of CD3 Spark Blue expression pre normalization with Cytonorm method (left) and post normalization (right).

In our own dataset, we measured control and validation samples in each batch. **Figure 4A** shows plots of control samples measured on several days with the CD3 marker. A density plot of all markers of the control samples in the multiple batches can help to identify markers that are sensitive to batch effects. The Cytonorm method to correct for batch effects can align signal variability with the use of control and validation samples (27). The Cytonorm method clusters the control samples of the different batches with FlowSOM clustering. The cell clusters found in control samples should not be affected by batch effects. Subsequently, the quantiles for each marker per cell cluster are computed and aligned. **Figure 4B** shows the effect of the Cytonorm method on a marker, such as CD3 Spark Blue. In the public dataset, FR-FCM-Z3WR, they randomly distributed their samples over the batches. Only visualizing their healthy control data also showed some differences between the batches and an effect on the FlowSOM clustering (https://github.com/HdBraanker/Spectral_Flow_Workflow/blob/main/supplementaryfile_Rscript.pdf).

### Subsampling

Typically, spectral flow cytometry experiments involve measuring many cells with many markers and sometimes many patients. To facilitate data exploration in large datasets, it is advised to subsample your data, saving computing time and memory, before analyzing the complete dataset. For subsampling two different methods can be used, either random selection of n number of cells (random downsampling), or density-dependent selection of n number of cells (density-dependent downsampling). We included a random downsampling function (*Downsampling_Flowset)* in our R script. Density-dependent downsampling is preferable when rare cell populations must be preserved, as it balances the cell distribution by either undersampling the major cell populations or oversampling the minor cell populations. Always test different number of cells for clustering ranging from 20.000 to 200.000 in total, since several studies found that sample size can have a major impact on clustering and cell population discovery (9, 28). Alternatively, to down sampling all samples, you can also first split your data into smaller parts, also called a training dataset and a larger test set. The small training dataset can be used to find the right clustering algorithm and number of clusters, before using the full dataset. A function to split your data is included in our script (*Subsampling_Flowset)*. This method allows more cells to be included, than downsampling. After data exploration with subsampled data, all data can also be used to generate final results. However, powerful computer hardware should be available.

### Exploring Data With Clustering and Dimensionality Reduction

With the number of clustering algorithms and dimension reduction methods available for flow cytometry it is difficult to choose the right clustering algorithm or dimensionality reduction method for your data. Several studies compared different cluster and dimensionality reduction methods for flow cytometry (28, 29). FlowSOM is one the fastest and best clustering algorithms for large flow cytometry datasets and is widely used (11). Commonly used dimensionality reduction methods are uniform manifold approximation and projection (UMAP) and t-distributed stochastic neighbor embedding (tSNE) (7, 9).

Different packages in R can be used to implement these algorithms. Before starting your analysis, setting a seed is recommended, because most algorithms are stochastic. Different runs can produce different results. By setting a seed, you can prevent different results and ensure reproducibility. However, first varying the seed can also help to explore the robustness of the results of an algorithm.

**Figure 5:**
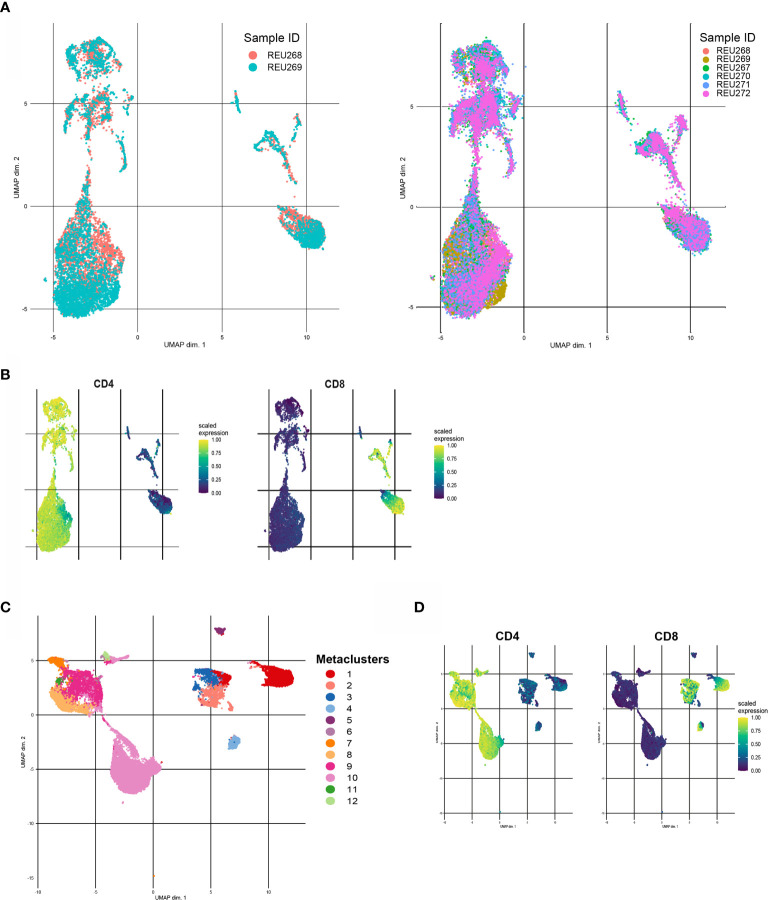
Different examples of clustering with FlowSOM and UMAP. **(A)** Unsupervised UMAP generated with a training set of the data (left) and UMAP with embedded data of the test set of the data (right). **(B)** CD4 (left) and CD8(right) scaled median expression in embedded UMAP. **(C)** UMAP of downsampled healthy control data (n=6) with cluster labels generated by FlowSOM clustering and metaclusters of Consensus Cluster Plus package. **(D)** CD4 (left) and CD8 (right) scaled median expression in UMAP.

Before clustering, we will start with dimensionality reduction methods to explore the different cell populations in our dataset. Additionally to the seed, various parameters can affect the results of an UMAP or tSNE. For example, the number of neighbors parameter in an UMAP controls how the algorithm balances local and global structure in the data. In the tSNE algorithm this is controlled by the perplexity parameter. Playing around with different values for these and other parameters in a subsampled dataset is advised to find the most optimal settings. **Figure 5A** shows an UMAP with optimized parameters of a subsampled training set of our data. The remaining test dataset can later be embedded to the UMAP. **Figure 5B** shows that for example CD4 and CD8 cell populations are nicely separated. Plotting expression of other markers in the UMAP will give insight in the present and number of cell populations in the dataset.

Next, clustering with for example FlowSOM can further help to interpret the data and statically test differences between groups, such as healthy controls versus patients or subgroups of patients. The CATALYST package provides functions to first cluster flow cytometry data with FlowSOM clustering and Consensus ClusterPlus package and visualize the metaclusters in an UMAP or tSNE (30). **Figure 5C** shows an UMAP with metacluster labels from the FlowSOM algorithm in our own dataset. **Figure 5D** shows that CD4 and CD8 cell populations also cluster differently with the FlowSOM algorithm. Subsequently, heatmaps of median expression per cluster and number of cells per cluster and/or group can be used to interpret the results. The diffcyt package can assist with performing statistical tests between different groups and/or metaclusters (31).

Determining the optimal number of clusters to use can be a challenge. A delta area plot or elbow plots are mathematical methods to determine the optimal number of clusters. First exploring data with unsupervised UMAP or tSNE before using clustering methods can also help to have idea of number of clusters in the data.

Alternatively to using R for clustering and dimensionality reduction, Cytosplore can be used (12). Cytosplore is interactive software and there is no need for advanced knowledge of R. Cytosplore offers different methods, such as SPADE clustering algorithm, approximated t-SNE (faster version of standard tSNE), a hierarchical SNE (hSNE). It can produce heatmaps of median expression per cluster and per group. Data or separate clusters can also be exported and further processed in R with the Cytofast package (32). Also, the Spectre R package, can be used. This package provides end-to-end workflow for R, can be adapted to work with spectral flow cytometry data and offers different cluster and dimensionality reduction methods (16).

## Discussion

With this article, we provide a step-by-step workflow on how to handle spectral flow cytometry datasets, that can be implemented by spectral flow cytometry users with basic R experience. First, manual data cleaning and quality control steps are described, followed by automated data cleaning. Next, data from different batches is normalized, preparing the data for automated dimensionality reduction or clustering analyses of choice. Following these preprocessing steps, downstream analysis results are more reproducible, due to the removal of technical artefacts and batch effects. The R code will be available on github and guides the user through pipeline with our own data and a publically available dataset (FR-FCM-Z3WR). Additional support can be asked *via* the Github page.

Compared to conventional flow cytometry, spectral flow cytometry has many advantages, such as use of larger panels, minimal compensation requirements and reducing autofluorescence effects. Also, practical aspects, such as reuse of reference controls, can save valuable time. Although mass cytometry also allows for large multicolor panels, acquisition time per sample can be long and many cells per sample are needed. Spectral flow cytometry has comparable acquisition times to conventional flow cytometry (4). In general, spectral flow cytometry is more comparable to conventional flow cytometry making it an attractive new technique for labs already experienced with conventional flow cytometry.

A large difference with conventional flowcytometry is the unmixing of the data after acquisition in spectral flow cytometry. In this workflow, raw spectral flow cytometry data were unmixed using SpectroFlo^®^ software. The method of unmixing spectral flow cytometry data can affect fluorescence intensity per cell. The SpectroFlo^®^ software uses linear unmixing, but other methods are also available. Novo et al. proposed that Poisson unmixing is the most accurate method of unmixing (33). However, we carefully optimized our panel to minimize unmixing artifacts with linear unmixing. Furthermore, we hope this article is a handle for starting researchers and implementing other unmixing methods requires advanced programming skills. Nevertheless, even if the unmixing method is changed, the other steps of our workflow can be used.

An arcsinh transformation can be used to transform spectral flow cytometry data and homogenize variances between cell populations for improved clustering results. A fixed cofactor can be used if fast exploration of the data is needed. Alternatively, the flowVS package or FlowTrans package can be used to calculate cofactors per fluorochrome (22, 23). Carefully inspect plots after data transformation is advised, because transformation artefacts, such as artificial extra spikes, should be avoided. Equally important to data transformation is correcting for or be aware of batch effects. The Cytonorm method was able to remove almost all batch effects in our data (27). We did not explore other packages to correct for batch effects, such as the integration of multibatch cytometry datasets (iMUBAC) package (34). The biggest advantage of the iMUBAC method is the independence of a control sample measured in all batches. Instead, it identifies common clusters between healthy controls in each batch and flags new clusters as batch effects. This method could stimulate research groups around the world to use the same flow cytometry panel and integrate their data. However, if a technical replicate is possible, the Cytonorm method is more stable and flexible. In our public dataset, the measured groups were randomly distributed over the batches. This can also reduce the influence of batch effects on the outcomes.

In most spectral flow cytometry experiments many cells and many markers will be measured. Depending on the research question, either discovering new cell populations or measuring differences in marker expression between populations, the method of subsampling can be chosen for downstream analysis. We proposed to use random downsampling or to split the dataset in smaller sets. Both methods are suitable to explore the data and find the best algorithm for your data and question. However, random downsampling can result in loss of very small cell populations and in that case density-dependent downsampling could be preferable (35). Furthermore, there is no clear guideline on number of cells to use per sample for dimension reduction methods. Liu et al. show that for clustering methods the performance and stability can vary with the number of cells used (28).

Many different clustering methods and dimension reduction techniques are available for flow cytometry data (29). In this paper, we only show the use of UMAP and FlowSOM. For flow cytometry, FlowSOM clustering seems to be the best performing algorithm (11, 28). Furthermore, we prefer UMAP to explore our data and visualize marker expression between samples or conditions, because it preserves more structure of the data. Since use of R for data analysis and exploration can be challenging, also software, such as Cytosplore can be easier-to-use if you start with spectral flow cytometry.

Adopting more standard workflows for (spectral) flow cytometry data would improve reproducibility of results. If additionally standardized panels would be used, data integration of patients from example multiple diseases or multiple countries would be possible and increase statistical power to find differences. However, this will be a major challenge and far from easy to accomplish for our field. Sharing more flow cytometry data in public flow cytometry repositories could, however, help to test new techniques for data integration and analyze data multiple times by different groups to catalyze new discoveries.

In conclusion, we present an easy-to-follow workflow to analyze spectral flow cytometry data for (new) researchers in the immunology field. Going through the workflow and optimizing the different steps will take one to two days. After preparing the data with the pipeline, additional time should be invested to do further data analysis and statistical testing. Standardizing spectral flow cytometry workflows will help to produce reproducible results and compare results between labs. Although our workflow is developed for spectral flow cytometry data, it could also be used for conventional and mass cytometry data. Our workflow is flexible and future developments can be easily integrated in the workflow helping starting researchers to obtain reproducible results in spectral flow cytometry.

## Data Availability Statement

Code used for data analysis can be found at https://github.com/HdBraanker/Spectral_Flow_Workflow. Publicly available datasets were analyzed in this study. These data can be found here: https://flowrepository.org/id/FR-FCM-Z3WR and https://flowrepository.org/id/FR-FCM-Z4KT. Further inquiries can be directed to the corresponding author.

## Ethics Statement

The studies involving human participants were reviewed and approved by the Erasmus MC medical ethics review board. The patients/participants provided their written informed consent to participate in this study.

## Author Contributions

HB and MB drafted the work, analyzed the data and wrote the manuscript. HB and MB have contributed equally to this work and share first authorship. EL critically revised the text and figures. All authors contributed to the article and approved the submitted version.

## Funding

Dutch Arthritis Association NR 19-2-401.

## Conflict of Interest

The authors declare that the research was conducted in the absence of any commercial or financial relationships that could be construed as a potential conflict of interest.

## Publisher’s Note

All claims expressed in this article are solely those of the authors and do not necessarily represent those of their affiliated organizations, or those of the publisher, the editors and the reviewers. Any product that may be evaluated in this article, or claim that may be made by its manufacturer, is not guaranteed or endorsed by the publisher.

## Supplementary Material

Supplemental File 1 Manual gating strategy.
