# Supplementary figures and images for: How to Prepare Spectral Flow Cytometry Datasets for High Dimensional Data Analysis: A Practical Workflow
Source: Front Immunol. 2021 Nov 19;12:768113. doi: 10.3389/fimmu.2021.768113 (PMC8640183; doi:10.3389/fimmu.2021.768113)

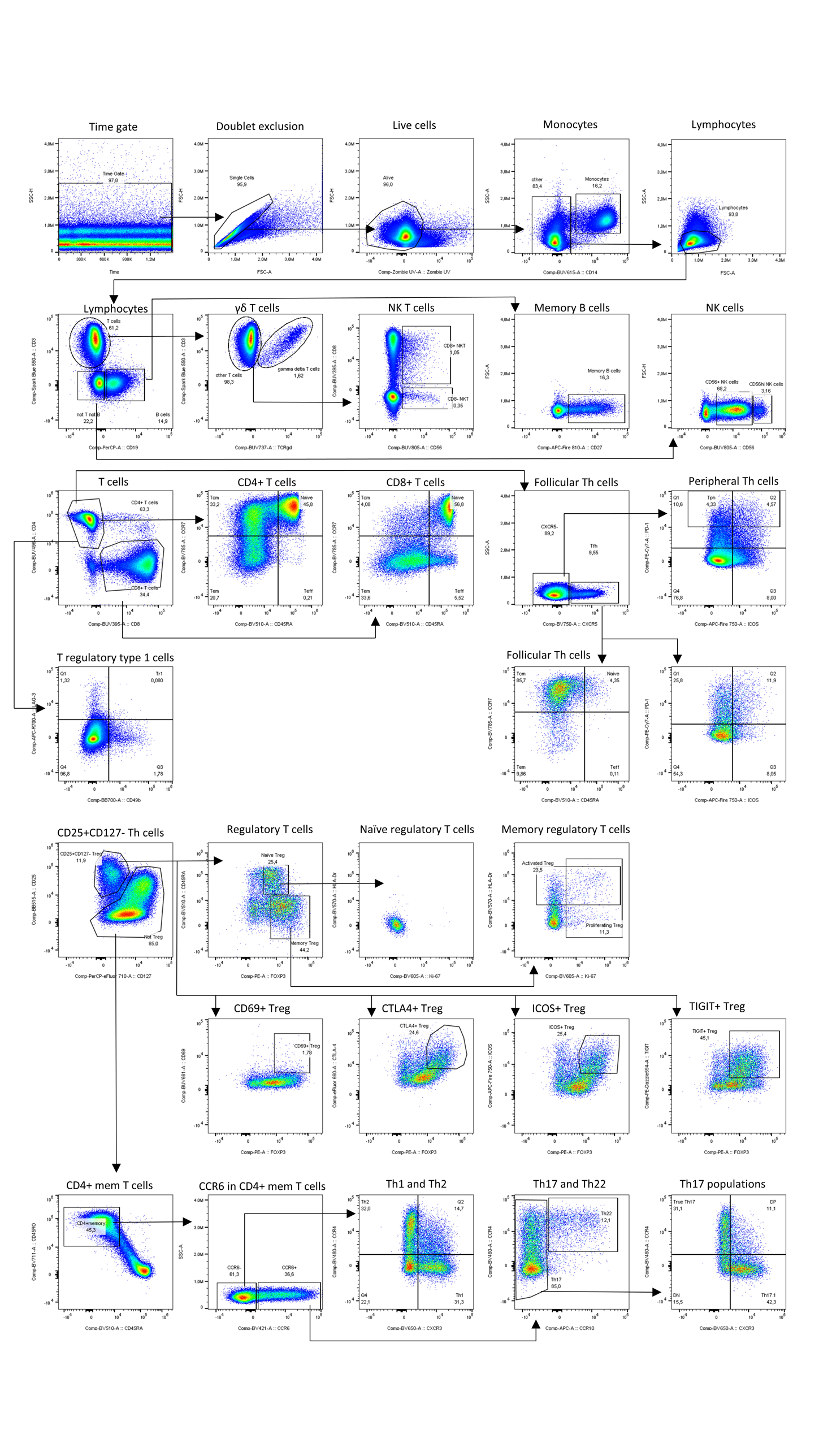

Supplement: Supplemental File 1 — Manual gating strategy. [file Image_1.jpeg]
